# From detection to intervention, optimizing care for patients with alcohol use disorder and advanced hepatic fibrosis

**DOI:** 10.1111/acer.15473

**Published:** 2024-10-27

**Authors:** Paola Zuluaga, Suthat Liangpunsakul

**Affiliations:** ^1^ Universitat Autònoma de Barcelona Bellaterra Spain; ^2^ Departament of Internal Medicine Hospital Universitari Germans Trias i Pujol Badalona Spain; ^3^ Division of Gastroenterology and Hepatology, Department of Medicine and Biochemistry Indiana University School of Medicine Indianapolis Indiana USA; ^4^ Division of Gastroenterology and Hepatology, Department of Molecular Biology Indiana University School of Medicine Indianapolis Indiana USA; ^5^ Roudebush Veterans Administration Medical Center Indianapolis Indiana USA

Nearly half of the world's population consumes alcohol, with approximately 20% engaging in binge drinking and 5%–10% drinking excessively over the long term (Manthey et al., 2019). Alcohol consumption is a leading cause of liver disease and mortality in both the United States and Europe, contributing to a significant public health burden (Karlsen et al., 2022). The risk of developing alcohol‐associated liver disease (ALD) varies widely among individuals, influenced by factors such as genetics (Schwantes‐An et al., 2024; Yuan et al., 2024), socioeconomic status (Askgaard et al., 2021), and specific drinking patterns (Aberg et al., 2017). Additionally, the stigma surrounding heavy drinking complicates accurate tracking of alcohol intake, leading to underreporting and misdiagnosis (Schomerus et al., 2022). Unlike many other liver diseases, ALD is often diagnosed late, typically after serious complications from portal hypertension or advanced fibrosis have developed (Shah et al., 2019).

The progression of liver disease in heavy drinkers is heterogeneous; while some individuals develop severe liver problems rapidly, others may experience a slower progression or remain relatively unaffected for years (Israelsen et al., 2024). Noninvasive tests (NITs) play a crucial role in identifying high‐risk patients who require intervention, distinguishing them from those at lower risk (Israelsen et al., 2024). Early detection through NITs focuses on two critical scenarios: screening at‐risk individuals for significant liver fibrosis and diagnosing or predicting outcomes for patients with advanced liver disease (Israelsen et al., 2024). Liver fibrosis, a key predictor of liver failure and mortality in asymptomatic ALD patients, can be assessed using various methods (Israelsen et al., 2024). These include elastography‐based tools such as liver stiffness measurement (LSM) and blood‐based markers such as the fibrosis‐4 test (FIB‐4) (Israelsen et al., 2024). While LSM provides valuable insights into liver stiffness, its availability is limited, particularly in nonspecialist settings, which restricts its use for routine screening and early detection of fibrosis (Israelsen et al., 2024). In contrast, blood‐based biomarkers such as the FIB‐4 test may offer a more convenient and accessible option for screening, allowing for easier implementation in various healthcare settings (Israelsen et al., 2024).

Patients with excessive alcohol use (EAU) or alcohol use disorder (AUD) represent a critical population for screening for advanced fibrosis and ALD, primarily due to their significantly elevated risk of liver damage. Prolonged heavy drinking can lead to the development of liver fibrosis, rendering AUD patients particularly susceptible to more severe liver diseases. Our recent study has highlighted the urgency of this issue, demonstrating that approximately one in five patients admitted for treatment of AUD exhibits FIB‐4 values indicative of advanced liver fibrosis (Zuluaga et al., 2024). Screening for liver fibrosis is essential, as it enables the identification of fibrosis at its initial stages, which can lead to timely interventions and significantly reduce the risk of serious complications.

In the October 2024 issue of *ACER*, Houston et al. (2024) examined whether patients with EAU and high FIB‐4 scores are being appropriately referred to hepatology. Analyzing records from 1131 patients in a large public health system between 2013 and 2023, they found that only 37% of the 316 patients with active EAU and high FIB‐4 scores were referred to hepatology (Houston et al., 2024). Patients with alcohol‐related mental health issues and those admitted for trauma were less likely to receive referrals, while those with alcohol‐related liver hospitalizations and higher comorbidity scores were more likely to be referred (Houston et al., 2024). Alarmingly, nearly 63% of patients at risk for advanced liver disease are not being referred for the care they need (Houston et al., 2024).

Screening for advanced fibrosis among patients with EAU or AUD is a valuable, but its effectiveness depends on the actions taken following the screening results. Identifying patients at risk for advanced fibrosis is just the initial step; it is essential for healthcare providers to implement appropriate follow‐up actions to address these findings effectively. The success of screening initiatives relies on a coordinated approach that emphasizes not only the detection of at‐risk individuals but also the importance of timely and appropriate clinical responses. This includes making prompt referrals to hepatology specialists, developing comprehensive management plans, and implementing targeted interventions tailored to each patient's specific needs. Without these follow‐up actions, screening efforts may fail to translate into meaningful improvements in patient outcomes.

According to the study by Houston et al. (2024), despite the awareness that patients with EAU and high FIB‐4 scores are at significant risk for advanced liver disease, referral rates to appropriate hepatology services remain alarmingly low. This trend is particularly pronounced among individuals with alcohol‐related mental health issues and those admitted for trauma, who frequently miss out on essential evaluations and referrals (Houston et al., 2024). Several factors may contribute to this concerning issue. First, healthcare providers often prioritize immediate mental health needs or trauma management over potential liver issues, especially in emergency or inpatient settings. This acute focus on psychiatric or physical crises can overshadow the importance of assessing underlying liver fibrosis (Johnson et al., 2022). As a result, the critical need for comprehensive evaluations of liver fibrosis may be overlooked, leading to missed opportunities for timely referrals. Second, mental health and primary care providers may be unfamiliar with noninvasive screening tools such as the FIB‐4 score and the specific liver‐related risks associated with alcohol consumption (Johnson et al., 2022). This knowledge gap can result in an underestimation of the severity of liver disease and a failure to recognize the necessity for specialized care. Third, the stigma surrounding mental health conditions can introduce biases into clinical care (Ahad et al., 2023). Providers might unconsciously view patients with alcohol‐related mental health issues as having less urgent medical concerns, which diminishes the emphasis on conducting essential screenings for liver disease. In contrast, patients with alcohol‐related liver hospitalizations typically present with clear and acute liver complications, making it more likely for healthcare providers to recognize the need for referrals. Finally, systemic issues such as fragmented care models and inadequate communication between primary care, mental health, and hepatology services further exacerbate the challenges in facilitating timely referrals (McGinty & Daumit, 2020; Zuchowski et al., 2015).

To enhance patient care in screening for advanced fibrosis among individuals with EAU or AUD within primary care and psychiatric settings, a multifaceted approach is essential. This begins with increasing education and training for providers on liver health, emphasizing the pathophysiology of ALD and the critical importance of early detection using NITs such as the FIB‐4 score. Implementing integrated care models that foster collaboration between primary care or mental health providers and hepatology services is vital for comprehensive management (Winder et al., 2022). Such models facilitate regular communication and the development of coordinated care plans, ensuring that both mental health and underlying advanced fibrosis are prioritized (Winder et al., 2022). Additionally, developing standardized screening protocols for routine liver assessments during primary care or psychiatric visits can streamline the identification of at‐risk individuals. Utilizing electronic health records to flag patients who require timely liver assessments will further enhance the screening process (Khan et al., 2017). Engaging patients through education about the importance of liver disease and the risks associated with EAU is paramount. Empowering patients to advocate for their well‐being can lead to greater involvement in their health care and encourage them to seek regular screenings. Establishing a robust system for timely follow‐up on screening results and referrals to specialty care is crucial for improving patient outcomes (Seyed‐Nezhad et al., 2021). This includes developing protocols for tracking screening results and facilitating communication among healthcare providers, ensuring that patients receive appropriate care without unnecessary delays (Sheehan et al., 2021). Furthermore, implementing a continuous quality improvement framework can monitor the effectiveness of screening and referral processes (Hill et al., 2020). By collecting and analyzing data on screening rates, referral patterns, and patient outcomes, healthcare providers can identify areas for improvement and inform ongoing training and policy adjustments (Hill et al., 2020).

The study by Houston et al. (2024) sheds light on the critical need for improved referral practices for patients with EAU and advanced fibrosis. By identifying the factors contributing to low referral rates to hepatology services, particularly among individuals with alcohol‐related mental health issues and trauma (Houston et al., 2024), this research underscores the importance of integrating liver health assessments into routine psychiatric and primary care. The insights gained from this study can inform targeted interventions, such as enhanced education for healthcare providers on the risks of ALD and the utilization of noninvasive screening tools. By addressing these gaps and fostering collaboration between primary care or mental health providers and hepatology services, we can improve the quality of care for patients with EAU and AUD, ensuring timely interventions and better health outcomes.

## CONFLICT OF INTEREST STATEMENT

None.
